# Recent Advances in Endometrial Cancer Management

**DOI:** 10.3390/jcm12062241

**Published:** 2023-03-14

**Authors:** Violante Di Donato, Andrea Giannini, Giorgio Bogani

**Affiliations:** Department of Gynecological, Obstetrical and Urological Sciences, “Sapienza” University of Rome, Italy-Policlinico Umberto I, Viale Regina Elena, 328, 00161 Roma, RM, Italy

In recent years, growing attempts have been carried out to improve the quality of care in the setting of gynecologic oncology, and, in particular, in endometrial cancer management [1]. Major advances included the introduction of even more sophisticated minimally invasive techniques and personalized treatments (including immune checkpoint inhibitors and targeted therapies) [2,3,4]. Major advances in the field of endometrial cancer are the use of sentinel node mapping through the use of florescence-guided minimally invasive surgery, the adoption of molecular and genomic profiling, and the introduction of immunotherapy [1,5,6]. Sentinel node mapping has replaced lymphadenectomy in the surgical staging of endometrial cancer [7,8]. Accumulating evidence highlighted that sentinel node mapping is accurate in identifying nodal involvement in low- and high-risk patients [7,8]. Data comparing sentinel node mapping, sentinel node mapping plus backup lymphadenectomy, and lymphadenectomy alone highlighted that sentinel node mapping is not inferior to conventional lymphadenctomy in identifying patients with nodal disease [7,8]. Sentinel node mapping in combination with ultrastaging allows the identification of more patients (twofold increase, approximately) with disease harboring in lymph nodes in comparison to conventional lymphadenectomy [9]. Ultrastaging allows the identification of isolated tumor cells and approximately 50% of micrometastasis not detectable via conventional histopathological analyses [9]. Although no specific data are available on the management of low-volume disease, the detection of isolated tumor cells and micrometastases provides important data regarding prognostication [10]. Furthermore, the introduction of molecular/genomic profiling would be useful in tailoring the most appropriate treatments [1,3]. In the advanced/metastatic setting, molecular characterization provides important information regarding the most appropriate treatments. MMRd/MSI-H is an important (agnostic) biomarker. Approximately, 30% of endometrial cancer patients harbor this type of alteration. Patients characterized by a ultra-mutated (*POLE*) and hyper-mutated (MSI-H) profile are likely to respond to immune checkpoint inhibitors. The GARNET study shows the beneficial effect of dostarlimab monotherapy in MMRd/MSI-H in 2L+ endometrial cancer patients [11]. Similarly, the Study 309 (KEYNOTE-775) highlighted that the combination of immunotherapy and tyrosine kinase inhibitor (i.e., pembrolizumab plus lenvatinib) provides a benefit in all patients [6]. Recently, ongoing studies have begun testing the use of immunotherapy in first-line (alone or in combination with chemotherapy) and maintenance settings (alone or in combination with PARP inhibitors). Moreover, exportin-1 inhibitor (e.g., selinexor) and WEE1 inhibitor seem to correlate with promising anti-tumor activity in TP53 wild type and TP53 mutated tumors [12]. In the era of personalized medicine, efforts are required to identify the best approach for every patient’s profile.

## References

[1] Jamieson A., McAlpine J.N. (2023). Molecular Profiling of Endometrial Cancer from TCGA to Clinical Practice. J. Natl. Compr. Cancer Netw..

[2] Bogani G., Dowdy S.C., Cliby W.A., Ghezzi F., Rossetti D., Frigerio L., Mariani A. (2016). Management of endometrial cancer: Issues and controversies. Eur. J. Gynaecol. Oncol..

[3] Cuccu I., D’Oria O., Sgamba L., De Angelis E., Golia D’Augè T., Turetta C., Di Dio C., Scudo M., Bogani G., Di Donato V. (2023). Role of Genomic and Molecular Biology in the Modulation of the Treatment of Endometrial Cancer: Narrative Review and Perspectives. Healthcare.

[4] Giannini A., Bogani G., Vizza E., Chiantera V., Laganà A.S., Muzii L., Salerno M.G., Caserta D., D’Oria O. (2022). Advances on Prevention and Screening of Gynecologic Tumors: Are We Stepping forward?. Healthcare.

[5] Burg L.C., Verheijen S., Bekkers R.L.M., IntHout J., Holloway R.W., Taskin S., Ferguson S.E., Xue Y., Ditto A., Baiocchi G. (2022). The added value of SLN mapping with indocyanine green in low- and intermediate-risk endometrial cancer management: A systematic review and meta-analysis. J. Gynecol. Oncol..

[6] Makker V., Colombo N., Casado Herráez A., Santin A.D., Colomba E., Miller D.S., Fujiwara K., Pignata S., Baron-Hay S., Ray-Coquard I. (2022). Lenvatinib plus Pembrolizumab for Advanced Endometrial Cancer. N. Engl. J. Med..

[7] Bogani G., Papadia A., Buda A., Casarin J., Di Donato V., Gasparri M.L., Plotti F., Pinelli C., Paderno M.C., Lopez S. (2021). Sentinel node mapping vs. sentinel node mapping plus back-up lymphadenectomy in high-risk endometrial cancer patients: Results from a multi-institutional study. Gynecol. Oncol..

[8] Ditto A., Martinelli F., Bogani G., Papadia A., Lorusso D., Raspagliesi F. (2015). Sentinel node mapping using hysteroscopic injection of indocyanine green and laparoscopic near-infrared fluorescence imaging in endometrial cancer staging. J. Minim. Invasive Gynecol..

[9] Bogani G., Mariani A., Paolini B., Ditto A., Raspagliesi F. (2019). Low-volume disease in endometrial cancer: The role of micrometastasis and isolated tumor cells. Gynecol. Oncol..

[10] Ghoniem K., Larish A.M., Dinoi G., Zhou X.C., Alhilli M., Wallace S., Wohlmuth C., Baiocchi G., Tokgozoglu N., Raspagliesi F. (2021). Oncologic outcomes of endometrial cancer in patients with low-volume metastasis in the sentinel lymph nodes: An international multi-institutional study. Gynecol. Oncol..

[11] Oaknin A., Gilbert L., Tinker A.V., Brown J., Mathews C., Press J., Sabatier R., O’Malley D.M., Samouelian V., Boni V. (2022). Safety and antitumor activity of dostarlimab in patients with advanced or recurrent DNA mismatch repair deficient/microsatellite instability-high (dMMR/MSI-H) or proficient/stable (MMRp/MSS) endometrial cancer: Interim results from GARNET-a phase I, single-arm study. J. Immunother. Cancer.

[12] Bogani G., Ray-Coquard I., Concin N., Ngoi N.Y.L., Morice P., Enomoto T., Takehara K., Denys H., Nout R.A., Lorusso D. (2021). Uterine serous carcinoma. Gynecol. Oncol..

